# *Mycoplasma hyopneumoniae* evades phagocytic uptake by porcine alveolar macrophages in vitro

**DOI:** 10.1186/s13567-019-0667-6

**Published:** 2019-06-24

**Authors:** Alannah S. Deeney, Gareth A. Maglennon, Ludivine Chapat, Steve Crussard, Edmond Jolivet, Andrew N. Rycroft

**Affiliations:** 1 Department of Pathobiology and Population Science, Royal Veterinary College, Hawkshead Lane, North Mymms, Hatfield, AL9 7TA UK; 2 Present Address: AstraZeneca UK Ltd., Cambridge Biomedical Campus, Cambridge, CB2 0AA UK; 3 Boehringer Ingelheim, Lyon, France

## Abstract

*Mycoplasma hyopneumoniae*, the agent of porcine enzootic pneumonia (EP), is able to persist in the lung tissue and evade destruction by the host for several weeks. To understand the mechanism of pathogen survival, phagocytic uptake of *M. hyopneumoniae* by primary porcine alveolar macrophages was investigated. Intracellular location and survival of the pathogen were explored using gentamicin survival assays, flow cytometry and confocal microscopy of *M. hyopneumoniae* 232 labelled with green fluorescent protein (GFP). Following 1 h and 16 h of co-incubation, few viable *M. hyopneumoniae* were recovered from inside macrophages. Flow cytometric analysis of macrophages incubated with *M. hyopneumoniae* expressing GFP indicated that the mycoplasmas became associated with macrophages, but were shown to be extracellular when actin-dependent phagocytosis was blocked with cytochalasin D. Confocal microscopy detected GFP-labelled *M. hyopneumoniae* inside macrophages and the numbers increased modestly with time of incubation. Neither the addition of porcine serum complement or convalescent serum from EP-recovered pigs was able to enhance engulfment of *M. hyopneumoniae*. This investigation suggests that *M. hyopneumoniae* evades significant uptake by porcine alveolar macrophages and this may be a mechanism of immune escape by *M. hyopneumoniae* in the porcine respiratory tract.

## Introduction

The porcine respiratory pathogen *Mycoplasma hyopneumoniae* is considered to be the primary agent of porcine enzootic pneumonia [1]. Enzootic pneumonia, a chronic disease, is a source of great economic loss worldwide because *M. hyopneumoniae*-infected animals exhibit reduced average daily weight gain and feed conversion [2, 3]. It has been shown that *M. hyopneumoniae* infection increases susceptibility of the pig to secondary infections which have further economic and welfare implications [4, 5]. At the time of slaughter, lung pathology arising from *M. hyopneumoniae* infection can be seen as regions of well-demarcated, dark purple, atelectatic tissue located in the anterior of the cranial and middle lung lobes, and apical anterior portion of the caudal lobe [6]. Microscopic inspection of lung lesions typically reveals loss of cilia from the respiratory ciliated epithelium, accumulation of lymphocytes and plasmocytes in peribronchial, peribronchiolar and perivascular areas, hyperplasia of bronchus associated lymphoid tissue, thickening of alveolar septae and exudate in bronchi and bronchiole lumens containing neutrophils and macrophages [7]. *M. hyopneumoniae*-induced lung lesions indicate the porcine host is producing an immune response to infection, but this response is not removing the pathogen quickly.

Management and control of enzootic pneumonia is achieved with good animal husbandry, disease surveillance and vaccination [8]. Many pig-producing countries practise vaccination against *M. hyopneumoniae* using adjuvanted inactivated whole-cell preparations, but live-attenuated vaccines are available in China and Mexico [8–10]. At present, all commercial *M. hyopneumoniae* vaccines offer reduced clinical and pathological signs of disease; however, they all fall short of providing complete protection from *M. hyopneumoniae* infection and the associated lung pathology. Hence, there is room to improve upon current *M. hyopneumoniae* vaccines [11].

In order to design and produce more effective vaccines against *M. hyopneumoniae*, better understanding of *M. hyopneumoniae* pathogenicity is required. A recent review by Maes et al. [10] recognised knowledge surrounding *M. hyopneumoniae* pathogenicity remains scarce but progress has been made. At present, lipoproteins at the surface of *M. hyopneumoniae* cells are recognised as adhesion proteins that facilitate attachment and colonisation of the ciliated epithelium [12, 13]. Some of these adhesion proteins are also thought to function as moonlighting proteins. For example, aminopeptidases at the cell-surface of *M. hyopneumoniae* bind to a number of different host molecules, and are also able to bind and cleave plasminogen to plasmin [14, 15]. The significance *M. hyopneumoniae*-directed plasmin activation in the in vivo environment and its role in disease pathogenesis remains to be determined. Speculative mechanisms of *M. hyopneumoniae* virulence include hydrogen peroxide production, modulation and evasion of host immunity [10]. More research is required to reveal pathogenicity mechanisms employed by *M. hyopneumoniae* to infect and colonise the porcine host.

During early infection and colonisation, *M. hyopneumoniae* is likely to encounter resident pulmonary phagocytes such as macrophages. Respiratory macrophages are an important first line of defence against invading microorganisms. They provide protection by engulfing and destroying susceptible organisms, they can also release chemical mediators to trigger an inflammatory immune response [16]. With this in mind, it is not clear from previous experimental data what role macrophages play in defence or propagation of *M. hyopneumoniae* infection. A number of pathogenic *Mycoplasma* spp. have demonstrated the ability to evade phagocytic uptake by host macrophages. For example, *M. pulmonis*, *M. bovis*, *M. dispar*, *M. pneumoniae* and *M. ovipneumoniae* all appear to resist uptake by host macrophages in the absence of specific opsonins. Upon addition of specific antisera these pathogenic mycoplasmas are readily engulfed [17–22]. It is possible that *M. hyopneumoniae* resists phagocytic uptake by respiratory macrophages, and this may in-part explain why *M. hyopneumoniae* is not quickly cleared by the respiratory immune system.

In this study, evasion of host immunity by *M. hyopneumoniae* was examined by investigating the interactions between *M. hyopneumoniae* and primary porcine alveolar macrophages. Experiments were designed to ascertain whether *M. hyopneumoniae* is phagocytosed by alveolar macrophages, and whether components of normal and convalescent porcine serum facilitate phagocytosis of *M. hyopneumoniae*.

## Materials and methods

### Bacterial strains and culture conditions

*M. hyopneumoniae* strain 232 was routinely cultured static at 37 °C in modified liquid Friis medium, and in a humidified atmosphere of 5% CO_2_ at 37 °C on modified Friis agar [1, 23–25].

*Escherichia coli* DH5α (Thermo Fisher Scientific, Massachusetts, USA), a K-12 derivative, was routinely cultured in LB broth [1% (w/v) tryptone (Oxoid, Hampshire, UK), 1% (w/v) NaCl and 0.5% (w/v) yeast extract (Oxoid)] at 37 °C with agitation at 210 rpm, and on LB agar [LB broth supplemented with 1.5% (w/v) bacteriological agar no. 1 (Oxoid)].

### Isolation and culture of primary porcine alveolar macrophages

Two conventional large white specific-pathogen free pigs were sourced from a herd which did not demonstrate viral-, enzootic pneumonia-, nor pleuropneumonia-like lung pathology at abattoir surveillance. The animals were euthanised by sedation with a ketamine and xylazine mixture, followed by overdose of sodium pentobarbital via ear vein injection. Primary alveolar macrophages were collected as done by Cullen, Rycroft [26]. Briefly, ice-cold sterile phosphate buffered saline (PBS) supplemented with 10 µg/mL gentamicin sulphate (Sigma Aldrich, Dorest, UK) was poured into the lungs, followed by gentle massage for 4 s and then the lavage fluid was collected into a sterile ice-cold glass bottle. The lungs from each animal appeared normal and did not present any macroscopic lesions suggestive of infection with respiratory pathogens such as *M. hyopneumoniae*, *Actinobacillus pleuropneumoniae* or porcine reproductive and respiratory syndrome virus. The lavage fluid was centrifuged at 200 × *g* for 10 min at room temperature. The cell pellet was resuspended in RPMI 1640 1 × medium (GIBCO, Thermo Fisher Scientific), supplemented with 10% heat-treated foetal bovine serum (FBS) (GIBCO). Viability counts were performed by trypan blue exclusion. If the number of viable cells was ≥ 90%, the cells were frozen on the day of procurement in FBS supplemented with 10% dimethyl sulfoxide (DMSO). These cells were identified as macrophages based on cellular morphology under the microscope and positive staining with anti-porcine CD-163 mouse antibody [27]. Two batches of alveolar macrophages were prepared, A and B, each batch originated from a different pig and were collected 1 week apart.

The alveolar macrophages were routinely cultured in RPMI 1640 Glutamax supplemented with HEPES (GIBCO) and 10% heat-treated FBS (referred to as tissue culture medium). The macrophages were maintained in vitro in 24-well plates in a humidified atmosphere of 5% CO_2_ at 37 °C for no more than 32 h.

### Plasmid construction

Polymerase chain reaction (PCR) was performed using Phusion High Fidelity DNA polymerase (NEB, Hertfordshire, UK) according to the manufacturers instructions. DNA oligonucleotides used in PCR amplification reactions are listed in Table 1. The plasmid pMHGFP-P97 was constructed by amplifying *M. hyopneumoniae* strain 232 *P97* promoter sequence from pMHC9-1 [28], using primers P97F and P97R, and was subsequently cloned into plasmid pMHGFP2. Plasmid pMHGFP2, constructed from pMHO-1 [29], contained the green fluorescent protein gene, *gfpmut2*, under control of the *spiralin* promoter sequence of *Spiroplasma citri* [28]; *gfpmut2* was amplified from the pKENmut2 vector [30] using primers gfpF and gfpR. The *spiralin* promoter sequence was digested from pMHGFP2, and replaced with the *P97* promoter sequence at restriction sites *Mlu*I and *Bam*HI to give rise to plasmid pMHGFP-P97.

**Table 1:** **DNA oligonucleotides**

Name	3′ sequence 5′
gfpF	TTACTAGTTGTGTGGAATTCGAGCTCGG
gfpR	TTGCATGCCTGCAGGTCTGG
P97F	GTCACGCGTCTTTTAATTATTAGTCTTCC
P97R	GTCGGATCCTTACTCATATTTTAAACCTC

Underlined nucleotides indicate restriction enzyme sequence

### Bacterial transformation

*E. coli* DH5α were transformed with ligation reactions by heat shock transformation. Briefly, ice-cold competent *E. coli* DH5α (100 μL) were incubated on ice for 30 min with ligation reactions (4 µL). *E. coli* were heat-shock transformed at 42 °C for 45 s and then cooled on ice for 2 min, followed by recovery in SOC medium (2% (w/v) tryptone (Oxoid), 0.5% (w/v) yeast extract (Oxoid), 0.05% (w/v) NaCl, 12.5 mM KCl, 10 mM MgCl_2_ and 20 mM glucose) (900 μL) for 1 h at 37 °C and 210 rpm. Transformed *E. coli* (100 μL) were spread on LB agar supplemented with 50 μg/mL ampicillin and incubated overnight at 37 °C.

*M. hyopneumoniae* strain 232 was transformed with plasmid DNA by electroporation as described previously [29]. Briefly, 25 mL of modified Friis medium inoculated with 0.5 mL of *M. hyopneumoniae* stock culture was grown static for approximately 41 h at 37 °C. The *M. hyopneumoniae* were washed and incubated on ice for 30 min with 10 μg of plasmid DNA. *M. hyopneumoniae* were transformed by electroporation at 2.5 kV, 100 Ω and 25 μF. Afterwards, 900 μL ice-cold Friis medium was added and the transformations were incubated on ice for 15 min. Transformed *M. hyopneumoniae* were allowed to recover for 3 h in static incubation, at 37 °C, and were then cultured on Friis agar supplemented with 0.2 μg/mL tetracycline at 37 °C in 5% CO_2_ for 7–14 days. Transformed *M. hyopneumoniae* colonies were transferred to 1 mL Friis medium supplemented with 0.5 μg/mL tetracycline, and incubated at 37 °C until the culture became peach/orange in colour. Liquid cultures at this stage were stored at −70 °C.

### Gentamicin survival assays

#### One hour gentamicin survival assay

Primary porcine alveolar macrophages (1 × 10^6^ in 1 mL) that had been frozen (batch A) were seeded into 24-well cell-culture treated plates (Nunc Nunclon Delta, Thermo Fisher Scientific) and allowed to adhere in a humidified atmosphere of 5% CO_2_ at 37 °C, for at least 1 h.

A *M. hyopneumoniae* culture (20 mL) grown for approximately 32 h was centrifuged, and then briefly washed in sterile PBS. The *M. hyopneumoniae* cell pellet was resuspended in 10 mL tissue culture medium. *E. coli* DH5α was grown to 1 × 10^8^ colony forming units (CFU)/mL, and 1 mL was centrifuged and washed in sterile PBS. The *E. coli* cell pellet was resuspended in 1 mL sterile PBS and then diluted 1 in 100 in tissue culture medium (10 mL).

Medium was removed from PAMs and replaced with either 1 mL of *M. hyopneumoniae* or *E. coli* inoculum, or tissue culture medium. The experiment was performed in duplicate and the intended multiplicity of infection (MOI) of bacteria/macrophage was 1:1. The inoculated PAMs were incubated in a humidified atmosphere of 5% CO_2_ at 37 °C for 1 h, and then the medium was removed and wells were washed very gently three times with sterile PBS. Tissue culture medium supplemented with or without 400 μg/mL gentamicin [31], was added to specified wells and the plate was incubated for a further 3 h. After incubation, culture supernatants of gentamicin-treated wells were collected and centrifuged at 16 000 × *g* for 2 min. Pellets were washed once in PBS, and then resuspended in 40 μL PBS and the entire volume was spread onto solid microbiological medium. The culture supernatants of wells without gentamicin were serially diluted 1 in 10 in PBS (1 mL) and 10 μL spots were inoculated onto solid microbiological medium.

After the culture supernatants had been removed, the wells were gently washed three times with warm sterile PBS. To wells inoculated with *M. hyopneumoniae* 400 μL of Friis medium was added, and 400 μL of LB broth to wells inoculated with *E. coli*. The well surfaces were scraped with sterile mini cell scrapers (Biotium, California, USA) and the contents were transferred to 2 mL microcentrifuge tubes containing a single tungsten carbide 3 mm bead (Qiagen, Manchester, UK). The tube contents were homogenised for 2 min at 30 cycles per second in a Retsch MM300 homogeniser (Retsch, Haan, Germany). It was previously confirmed that this method of homogenisation did not reduce the viability of *M. hyopneumoniae*. The homogenised samples that had not been treated with gentamicin, were serially diluted 1 in 10 in PBS (1 mL) and 10 μL spots were spotted onto solid microbiological medium. The entire volume (400 μL) of gentamicin-treated samples was spread onto 9 cm plates. Samples from uninoculated controls were plated to check for contaminating microorganisms.

#### Overnight gentamicin survival assay

Primary porcine alveolar macrophages (1 × 10^6^ in 1 mL) that had been frozen (batch A), were seeded into 24-well cell-culture treated plates and were incubated for at least 1 h.

*M. hyopneumoniae* and *E. coli* were prepared similarly to the 1 h gentamicin survival assay, except the inoculum was reduced such that the starting MOI of bacteria/macrophage was 1:100.

The medium was removed from macrophages and replaced with either 1 mL of *M. hyopneumoniae* or *E. coli* inoculum. The macrophages and bacteria were incubated static in a humidified atmosphere of 5% CO_2_ at 37 °C for approximately 16 h, and then tissue culture medium supplemented with or without 400 µg/mL gentamicin was applied for 6 h. After the first 3 h of incubation the tissue culture medium ± gentamicin was refreshed. The experiment was performed in triplicate.

### Flow cytometry

#### Flow cytometric analysis of GFP-transformed bacteria

Untransformed bacteria diluted 1 in 10 in PBS, were analysed on BD Accuri C6 flow cytometer (BD Bioscience, California, USA) to gate bacterial cells. GFP-transformed bacteria were also diluted 1 in 10 in PBS and analysed. Green fluorescent bacteria were detected by the FL1 detector. To distinguish fluorescent and non-fluorescent bacterial cells, a marker was drawn on the FL1 histogram plot of untransformed bacteria. This marker was then applied to FL1 histogram plots of GFP-transformed bacteria to establish the percentage of fluorescent bacteria.

#### Flow cytometric analysis of phagocytic uptake of GFP-labelled bacteria

Alveolar macrophages (5 × 10^5^ in 0.5 mL per reaction) that had been frozen (batch A) were supplemented with or without 20 μg/mL cytochalasin D (CCD) (Sigma Aldrich, catalogue C2618). The macrophages (0.5 mL) were seeded into wells of a 24-well plate and incubated for 15 min at 37 °C prior to the addition of bacteria.

A 200 mL *M. hyopneumoniae* (pMHGFP-P97) culture was prepared by inoculating 0.5 mL stock culture into 200 mL Friis medium. This culture was grown for 122 h until the medium was orange in colour. The *M. hyopneumoniae* (pMHGFP-P97) culture was centrifuged at 9000 × *g* for 10 min at room temperature. The mycoplasma pellet was briefly washed in sterile PBS, and then resuspended in 4 mL tissue culture medium such that 200 μL was equal to 25 mL original culture. *E. coli* (pKENmut2) was grown until optical density_600nm_ (OD_600nm_) 0.6, centrifuged at 2500 ×* g* for 5 min, washed briefly in PBS and then diluted to 1.3 × 10^7^ CFU/200 μL. The intended MOI of bacteria/PAM was 25:1. The macrophages, with and without CCD, were inoculated with 0.5 mL *M. hyopneumoniae* (pMHGFP-P97) or *E. coli* (pKENmut2); this halved the CCD concentration to 10 μg/mL. Some of the wells containing macrophages, with and without CDD, were mock-inoculated with tissue culture medium as the negative controls. The experiment was performed in triplicate. The macrophages were incubated with the bacteria for 1 h, static, in a humidified atmosphere of 5% CO_2_ at 37 °C. After incubation, the macrophages were gently scrapped from the well surface with mini cell scrappers, and then centrifuged at 400 × *g* for 5 min at room temperature in microcentrifuge tubes. The supernatants were removed and the macrophage cells pellets were resuspended in 500 μL FACSFlow. The macrophages were analysed immediately on a FACSCalibur flow cytometer (BD) using the FL1 detector. Uninoculated macrophages were used to gate non-fluorescent cells. The flow cytometric data were analysed in FlowJo version 10 software (FlowJo LLC, Oregon, USA).

### Confocal microscopy

#### Visualising GFP-transformed M. hyopneumoniae

Liquid cultures of GFP-transformed *M. hyopneumoniae* were grown until orange (72 h) in colour. The cultures were centrifuged at 9000 × *g* for 10 min at room temperature, and washed once in 1 mL PBS. *M. hyopneumoniae* cells were mounted onto glass coverslips as previously described [14]. Briefly, *M. hyopneumoniae* cells were resuspended in 100 μL PBS for every 1 mL of original culture. Glass coverslips (22 × 22 mm, Chance Propper, Warley, UK) were coated with 0.01% poly-l-lysine (w/v) (Sigma Aldrich) and air-dried at room temperature. *M. hyopneumoniae* suspensions (100 μL) were spotted onto the poly-l-lysine coated coverslips and allowed to adhere for 30 min at room temperature. The coverslips were washed twice in PBS, then *M. hyopneumoniae* were fixed in 2% formaldehyde in PBS overnight at 4–8 °C. Formaldehyde was removed by three brief washes in PBS. *M. hyopneumoniae* cells were stained with 1 μg/mL 4′,6-diamidino-2-phenylindole dihydrochloride (DAPI) (Roche Diagnostics, Mannheim, Germany) in PBS for 30 min at room temperature in the dark, followed by two brief washes with PBS. The coverslips were air-dried, and then 5 μL of Vectashield (Vector Laboratories, California, USA) was spotted on the coverslip and a glass slide was placed on top. The edges of the coverslip were sealed with transparent nail varnish (Maybelline, New York, USA).

GFP-transformed *M. hyopneumoniae* mounted onto glass coverslips were viewed on a confocal scanning laser Leica SP5 microscope (Leica Microsystems, Wetzlar, Germany). The samples were viewed by 63× objective with 4× digital zoom. Blue light (405 nm) was used to excite DAPI to visualise nucleic acid, and blue light (488 nm) to excite and visualise GFP. Untransformed *M. hyopneumoniae* were used as the negative control.

#### Confocal microscopy analysis of porcine alveolar macrophages infected with GFP-labelled *M. hyopneumoniae*

##### Time-dependent experiments

Primary porcine alveolar macrophages that had been frozen (batch B) were seeded (2.5 × 10^5^ cells per well) into an 8-chamber glass Lab-Tek Chamber Slide System (Nunc). The macrophages were incubated for at least 1 h prior to use.

A *M. hyopneumoniae* (pMHGFP-P97) culture that was orange in colour was centrifuged at 9000 ×* g* for 10 min at room temperature. The mycoplasma pellet was washed briefly in PBS and then resuspended in tissue culture medium (supplemented with 0.5 μg/mL tetracycline hydrochloride) such that 200 μL was equal to 12.5 mL of original culture. When *M. hyopneumoniae* wild type strain 232 were used, the mycoplasma pellet was suspended in tissue culture medium such that 200 μL was equal to 5 mL of original culture. *E. coli* (pKENmut2) were grown until OD_600nm_ 0.6. The *E. coli* were centrifuged at 2500 ×* g* for 5 min at room temperature, and briefly washed in PBS. The *E. coli* cell pellet was resuspended and diluted in tissue culture medium (supplemented with 50 μg/mL ampicillin) such that 200 μL contained 2.5 × 10^6^ CFU.

The tissue culture medium was removed from macrophages, and replaced with either 200 μL of *M. hyopneumoniae* (pMHGFP-P97), or *M. hyopneumoniae* wild type, or *E. coli* (pKENmut2). The macrophages and bacteria were incubated for the specified time in a humidified atmosphere of 5% CO_2_ at 37 °C. After incubation, the culture medium was removed from wells and each well was gently washed with warm PBS. The samples were fixed with 2% paraformaldehyde in PBS (Sigma Aldrich) overnight at 4–8 °C in the dark.

Following fixation, the wells were washed with PBS twice, and then 100 μL 0.5% Triton-X-100 in PBS was added, and the samples were incubated at room temperature for 15 min. The wells were then washed with PBS three times, and 5% bovine serum albumin in PBS was added and the samples were incubated at 4–8 °C for 30 min. The wells were then washed with PBS twice, and 200 μL of 1 μg/mL DAPI and 5 U/mL phalloidin-CF568 (Biotium) in PBS was added, and the samples were incubated at room temperature for 30 min in the dark. The wells were washed with PBS twice and then 200 μL of 1 μg/mL mouse IgG1 anti-GFP (Sigma Aldrich, catalogue SAB4600051) was applied to each well and incubated at 4–8 °C for 1 h in the dark. After this incubation, the wells were washed with PBS three times, and the chamber and gasket were removed from the slide. The slide was air-dried and 30 μL of Vectashield was spotted onto the slide. A glass coverslip was placed on top and the edges were sealed with transparent nail varnish.

The samples were viewed on a confocal scanning laser Leica SP5 microscope. The samples were viewed by 63× objective with 4× digital zoom. Blue light (405 nm) was used to excite DAPI to visualise nucleic acid, blue light (488 nm) to excite and visualise GFP, and yellow light (594 nm) to excite phalloidin-CF568 to visualise macrophage actin. Micrographs were captured at increments of 0.13 μm along the z-axis to produce z-stack images of macrophages. To determine if bacteria were inside macrophages, the z-stack micrographs were viewed in x, y and z-planes simultaneously using the computer software Volocity version 6.3 (PerkinElmer, Massachusetts, USA).

#### Opsonisation experiments

Opsonisation of *M. hyopneumoniae* (pMHGFP-P97) and *E. coli* (pKENmut2) was performed using preimmune porcine serum and convalescent porcine sera A, B and C. The preimmune and convalescent sera A were derived from the same pig at different times; the preimmune serum was collected before *M. hyopneumoniae* infection and serum A was collected 5 weeks after infection with *M. hyopneumoniae* strain 232 (supplied by Jessica Beddow). Serum B was derived from a pig infected with *M. hyopneumoniae* strain 325 (known as 9954 serum), and serum C was derived from a pig infected with *M. hyopneumoniae* strain 89 (known as 9965 serum) (supplied by Andrew Rycroft). The presence of *M. hyopneumoniae*-specific antibody in the convalescent sera was confirmed by Western Blot (data not shown). Heat-treated FBS was the negative control serum.

*M. hyopneumoniae* (pMHGFP-P97) cell pellets, each equivalent to 12.5 mL original culture, were resuspended in 100 μL of tissue culture medium supplemented with 1 μg/mL tetracycline hydrochloride. To separate *M. hyopneumoniae* (pMHGFP-P97) suspensions (100 μL), 100 μL of the different sera were added and incubated at 37 °C for 15 min. *E. coli* (pKENmut2) pellets, each equivalent to 2.5 × 10^6^ CFU, were resuspended in 100 μL of tissue culture medium supplemented with 100 μg/mL ampicillin sodium salt. These *E. coli* (pKENmut2) suspensions were treated the same as *M. hyopneumoniae* (pMHGFP-P97). Macrophages (batch B) were inoculated with these 200 μL suspensions of bacteria and incubated for 2 h. All other experiment details were identical to the time-dependent experiments.

## Results

### Few viable *M. hyopneumoniae* were recovered from inside porcine macrophages

**Figure 1:**
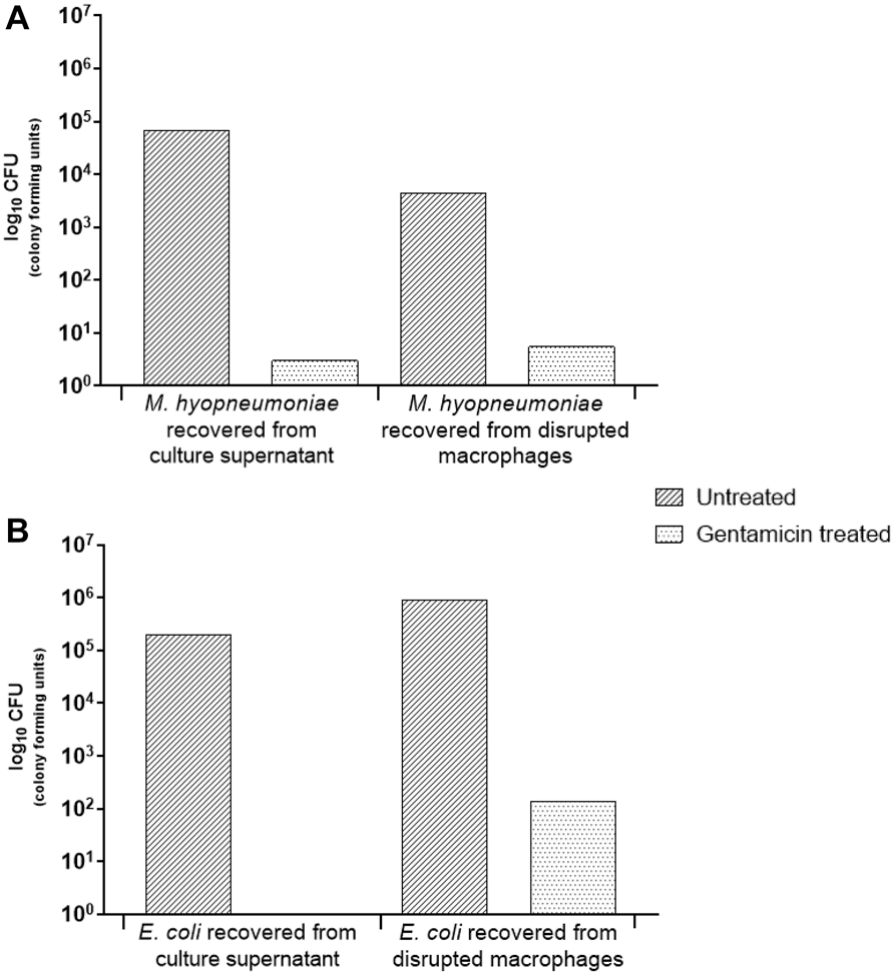
**1** **h phagocytosis assay of** ***M. hyopneumoniae.*** Porcine alveolar macrophages were incubated with *M. hyopneumoniae* or *E. coli* for 1 h and then 400 µg/mL gentamicin was applied for 3 h. Samples were plated on to solid medium to enumerate bacteria. **A** *M. hyopneumoniae* recovered from culture supernatant or disrupted macrophages, with and without gentamicin treatment. **B** *E. coli* recovered from culture supernatant or disrupted macrophages, with and without gentamicin treatment.

To evaluate the ability of porcine lung macrophages to engulf *M. hyopneumoniae*, cells were prepared by lavage of the lungs from a freshly killed pig known to be free of *M. hyopneumoniae* and other respiratory pathogens [26]. Primary porcine alveolar macrophages were incubated with either *M. hyopneumoniae* 232 or *E. coli* DH5α for 1 h before addition of gentamicin for 3 h to kill extracellular bacteria. Enumeration of bacteria in the cell supernatants showed complete destruction of the extracellular *E. coli*. However, a small proportion of *M. hyopneumoniae* (0.0045%) survived the treatment with gentamicin (Figure 1A). Internalised bacteria were then recovered by rupture of lung cells by mechanical homogenisation. Culture of the cell homogenate revealed 0.125% of the *M. hyopneumoniae* were viable compared to the control without antibiotic (Figure 1A). The effectiveness of gentamicin treatment and efficiency of macrophage phagocytic function was confirmed as no *E. coli* DH5α were recovered from supernatant but *E. coli* were recovered from the cell homogenate (Figure 1B).

**Figure 2:**
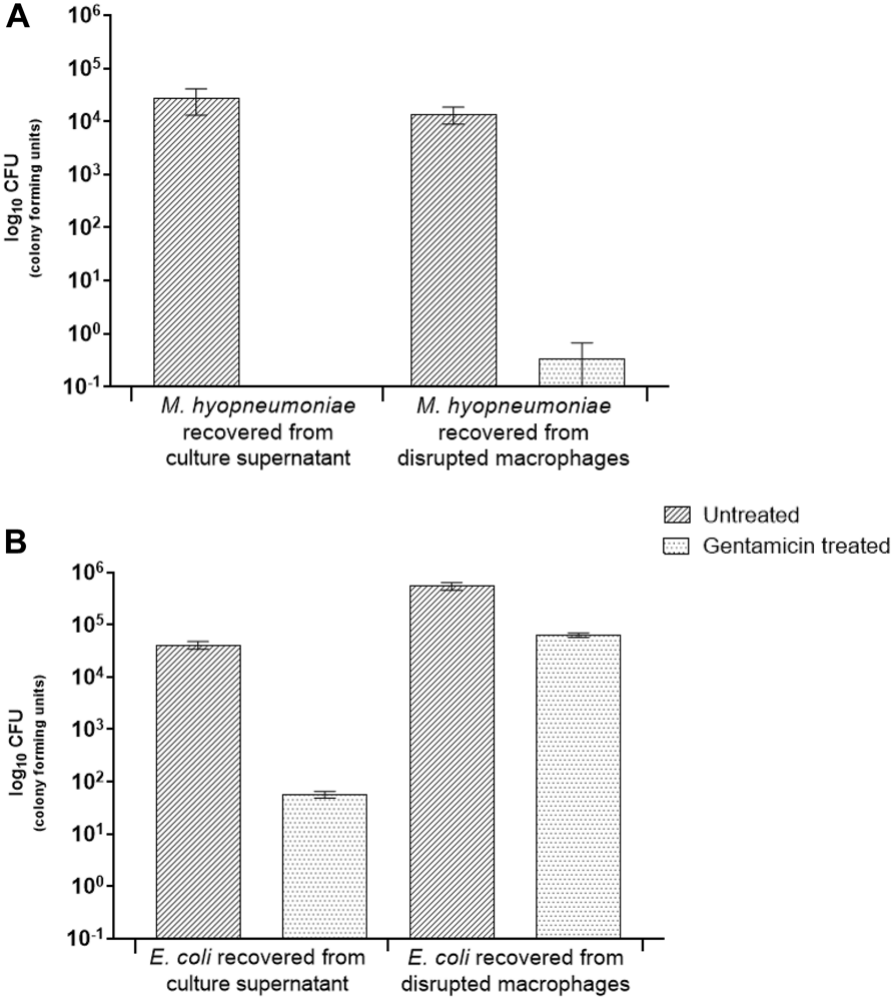
**Overnight phagocytosis assay of** ***M. hyopneumoniae.*** Porcine alveolar macrophages were incubated with *M. hyopneumoniae* or *E. coli* overnight (16 h) and then 400 µg/mL gentamicin was applied for 6 h. Samples were plated on to solid medium to enumerate bacteria. **A** *M. hyopneumoniae* recovered from culture supernatant or disrupted macrophages, with and without gentamicin treatment. **B** *E. coli* recovered from culture supernatant or disrupted macrophages, with and without gentamicin treatment. Error bars show standard error of the mean from one experiment performed in triplicate.

To determine whether phagocytic activity was time-dependent, *M. hyopneumoniae* were incubated with lung macrophages for 16 h. Gentamicin (400 μg/mL) was then applied for 6 h. Extended exposure to gentamicin was intended to eliminate all extracellular *M. hyopneumoniae.* No *M. hyopneumoniae* were recovered from the culture supernatants following extended exposure to gentamicin. This indicated that all extracellular *M. hyopneumoniae* had been eliminated (Figure 2A). No *M. hyopneumoniae* were recovered from gentamicin-treated disrupted macrophages while numerous viable *M. hyopneumoniae* were recovered from untreated macrophages (Figure 2A). This result suggested that very few *M. hyopneumoniae* had been phagocytosed by macrophages.

### Expression and detection of green fluorescent protein in *M. hyopneumoniae*

**Figure 3:**
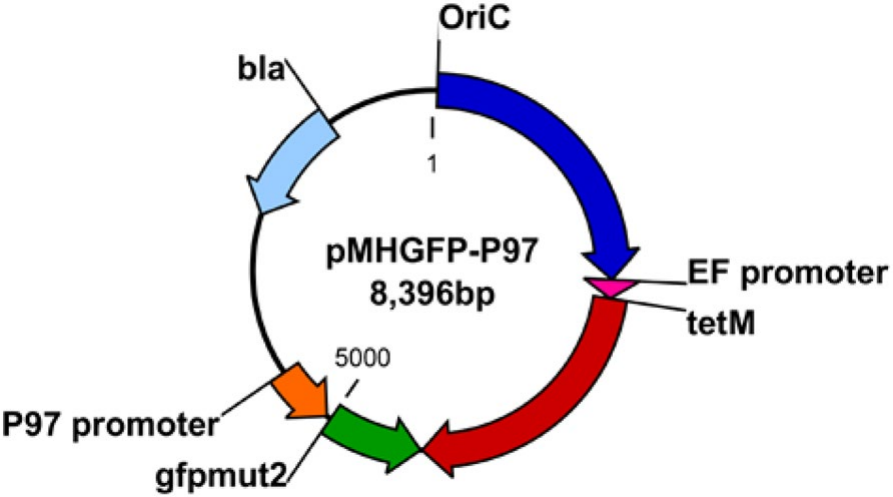
**pMHGFP-P97 plasmid diagram.** Oric = *M. hyopneumoniae* strain 232 predicited origin of replication, EF promoter = *tetM* gene promoter from *Enterococcus faecalis*, *tetM* = tetracycline resistance gene, *gfpmut2* = green fluorescent protein gene, *P97* promoter = predicted *P97* gene promoter from *M. hyopneumoniae* strain 232, *bla* = ampicillin resistance gene.

In order to express GFP in *M. hyopneumoniae*, a plasmid carrying the GFP gene *gfpmut2* was constructed from a plasmid known to be stably inherited in *M. hyopneumoniae*. The plasmid construct was based on pMHO-1 which contained the predicted origin of replication of *M. hyopneumoniae* strain 232, the *tetM* tetracycline resistance gene expressed under control of its natural promoter from *Enterococcus faecalis*, and the *bla* ampicillin resistance gene [29]. Plasmid pMHGFP-P97 was produced by cloning the *gfpmut2* gene under control of the *P97* gene promoter sequence, from *M. hyopneumoniae* strain 232, into plasmid pMHO-1 (Figure 3).

*M. hyopneumoniae* strain 232 was electroporated with plasmid pMHGFP-P97 and transformants were recovered on selective medium. Transformed *M. hyopneumoniae* were viewed using a confocal laser scanning microscope for green fluorescence.

**Figure 4:**
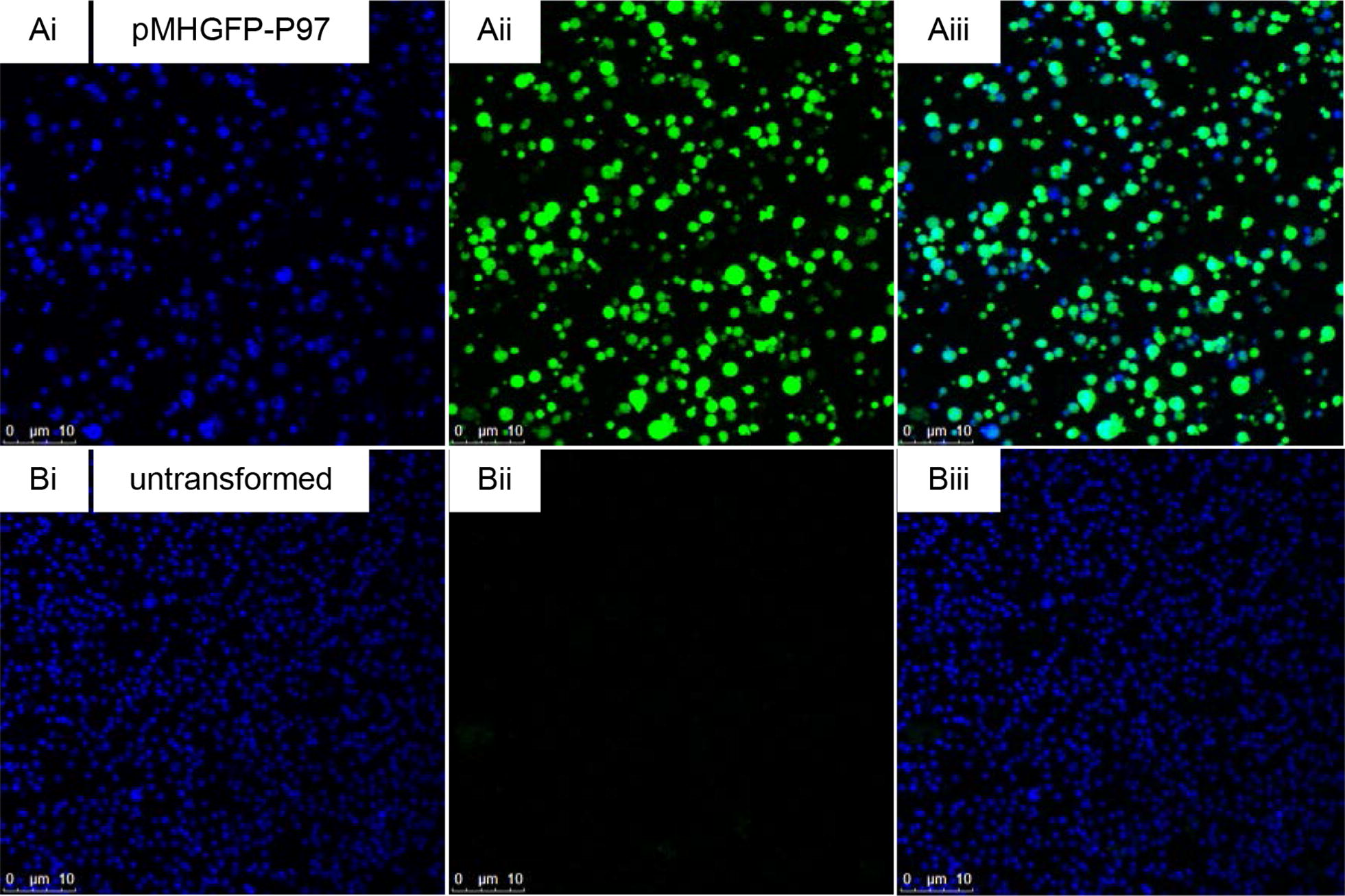
**Confocal micrographs of** ***M. hyopneumoniae*** **(pMHGFP-P97).** *M. hyopneumoniae* cells were stained with 4′,6-diamidino-2-phenylindole (DAPI) and were excited by 405 nm and 488 nm lasers to visualise nucleic acid and green fluorescence, respectively. **A** (i) *M. hyopneumoniae* (pMHGFP-P97) cells stained by DAPI, (ii) *M. hyopneumoniae* (pMHGFP-P97) cells viewed on green fluorescence channel, (iii) overlay of i and ii. **B** (i) untransformed *M. hyopneumoniae* cells stained by DAPI, (ii) untransformed *M. hyopneumoniae* cells viewed on green fluorescence channel, (iii) overlay of i and ii.

*M. hyopneumoniae* (pMHGFP-P97) cells fluoresced green, enabling visualization of individual mycoplasma cells (Figure 4).

**Figure 5:**
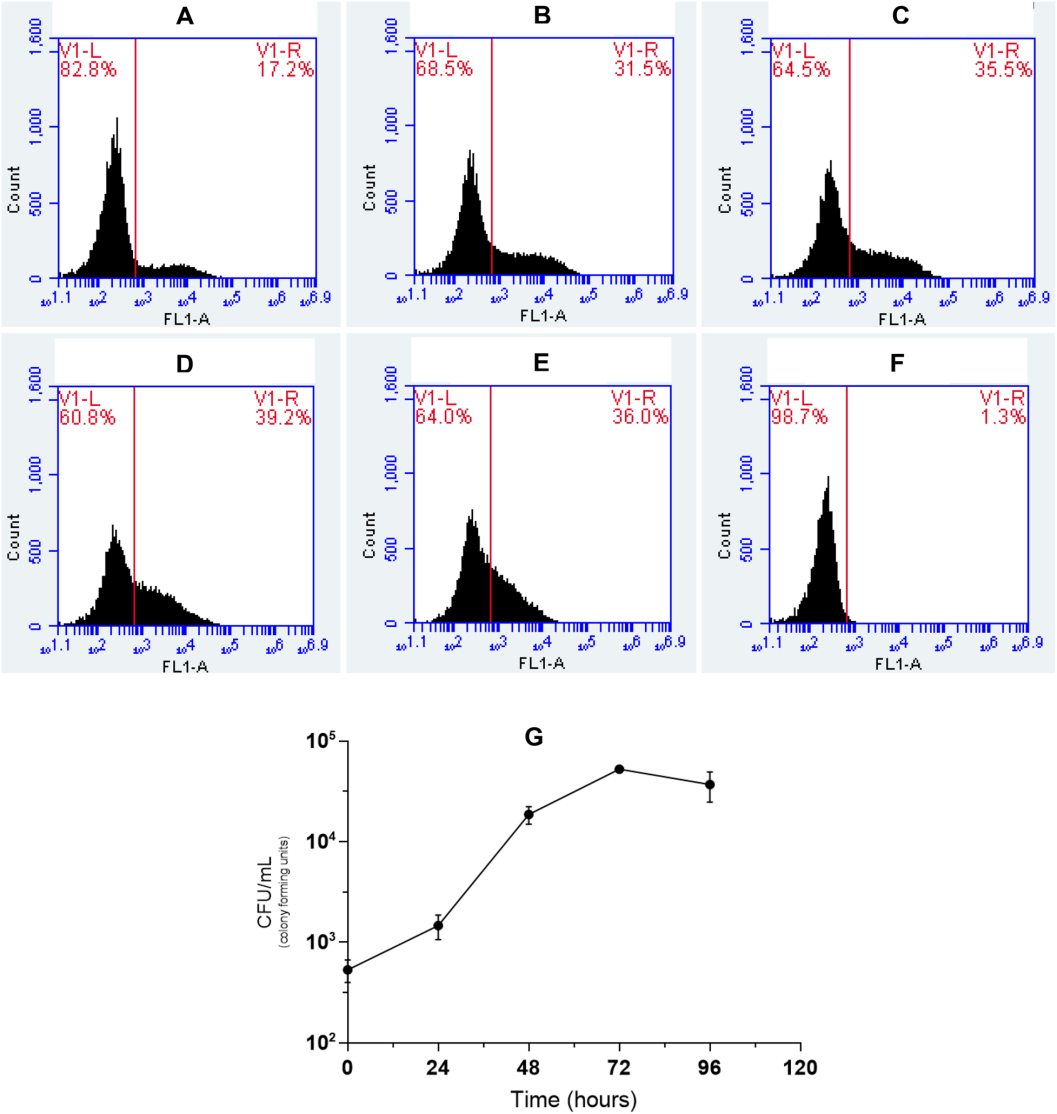
**Flow cytometric analysis of** ***M. hyopneumoniae*** **(pMHGFP-P97) fluorescence over time.** A *M. hyopneumoniae* (pMHGFP-P97) culture was analysed for green fluorescence over time by flow cytometry. The histogram FL1-A axis depicts green fluorescence intensity. On histograms plots, the vertical line indicates the boundary between fluorescent and non-fluorescent cells, to right of the line are fluorescent cells. **A** 24 h. **B** 48 h. **C** 72 h. **D** 96 h. **E** 168 h. **F** Untransformed *M. hyopneumoniae*. **G** Viable counts of *M. hyopneumoniae* (pMHGFP-P97) were performed from 24 to 96 h. Error bars show standard error of the mean.

*M. hyopneumoniae* (pMHGFP-P97) was analysed further analysis by flow cytometry. The effect of culture time on fluorescence intensity was determined. At each time point the fluorescent population was depicted by a broad peak suggesting that cells differed in fluorescence intensity. At 24 h post-inoculation, 17% of the *M. hyopneumoniae* (pMHGFP-P97) population were fluorescing green, this increased in intensity with incubation to 96 h (Figure 5). The intensity of fluorescence of *M. hyopneumoniae* (pMHGFP-P97) was considered sufficient as a label in further investigations.

### Flow cytometric analysis showed *M. hyopneumoniae* was not phagocytosed by macrophages

A flow cytometric phagocytosis assay using GFP-labelled *M. hyopneumoniae* 232 (pMHGFP-P97) was used to investigate phagocytic uptake by porcine alveolar macrophages [32, 33]. The assay determines phagocytic uptake of fluorescent bacteria by comparing the fluorescence intensity of phagocytes that have been treated with and without cytochalasin D (CCD), an inhibitor of actin-dependent phagocytosis. Uptake of fluorescent bacteria is evident when CCD-treated phagocytes have lower fluorescence intensity than the untreated phagocytes, because CCD-treated phagocytes only have externally-associated bacteria. Alveolar macrophages treated with CCD, or left untreated, were incubated with *M. hyopneumoniae* (pMHGFP-P97) for 1 h. Green fluorescent *E. coli* (pKENmut2) was used as the control organism.

**Figure 6:**
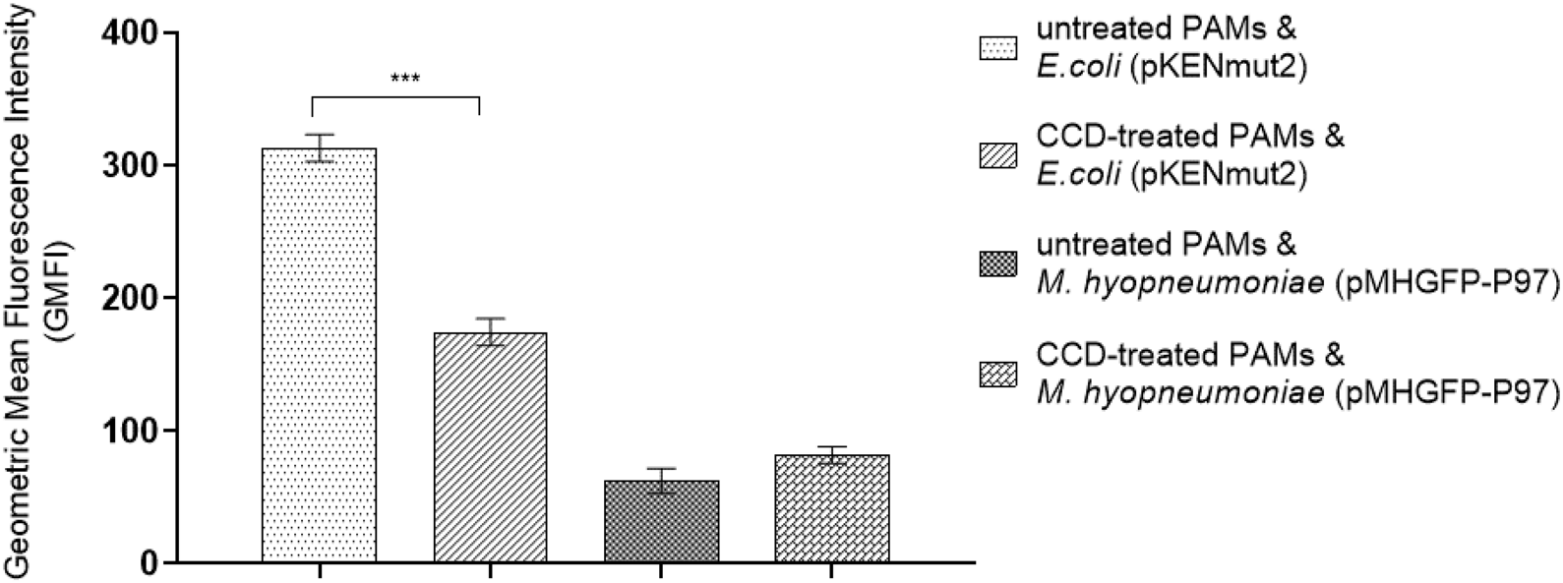
**Geometric mean fluorescence intensities of macrophages, without and without CCD, after incubation with GFP-labelled** ***M. hyopneumoniae*** **and** ***E. coli.*** The green fluorescence intensity of PAMs following incubation with GFP-labelled *M. hyopneumoniae* (*M. hyopneumoniae* (pMHGFP-P97)) or GFP-labelled *E. coli* (*E. coli* (pKENmut2)) was measured and normalised against uninoculated PAMs. Error bars show standard error of the mean from two separate experiments, 6 replicates in total. Significant mean differences were determined by independent T-test (PAMs ± CCD and *E. coli*) and Mann–Whitney U test (PAMs ± CCD and *M. hyopneumoniae*), ***p < 0.001. Porcine alveolar macrophages (PAMs), cytochalasin D (CCD).

Untreated macrophages incubated with the positive control *E. coli* had significantly higher fluorescence intensity than CCD-treated macrophages (Figure 6). This result confirmed untreated macrophages had taken up *E. coli* (pKENmut2), and that phagocytosis was inhibited by CCD. In contrast, the mean fluorescence intensity of macrophages incubated with *M. hyopneumoniae* (pMHGFP-P97), with and without CCD, were very similar (Figure 6). The fluorescence intensity of CCD-treated macrophages was marginally higher than untreated macrophages, but this difference was not significant. It was suggested from these results that more *M. hyopneumoniae* (pMHGFP-P97) were bound to CCD-treated macrophages than untreated macrophages. It was therefore concluded that *M. hyopneumoniae* (pMHGFP-P97) had not been engulfed by primary macrophages.

### Internalised *M. hyopneumoniae* detection by confocal microscopy

*M. hyopneumoniae* (pMHGFP-P97) was utilised in confocal microscopy experiments to look for evidence of uptake by porcine alveolar macrophages. Cells were incubated with *M. hyopneumoniae* (pMHGFP-P97) for 2, 4 and 6 h, and were examined for green-labelled mycoplasma localised in the cytoplasm. *E. coli* (pKENmut2) was again used as the positive control organism.

**Figure 7:**
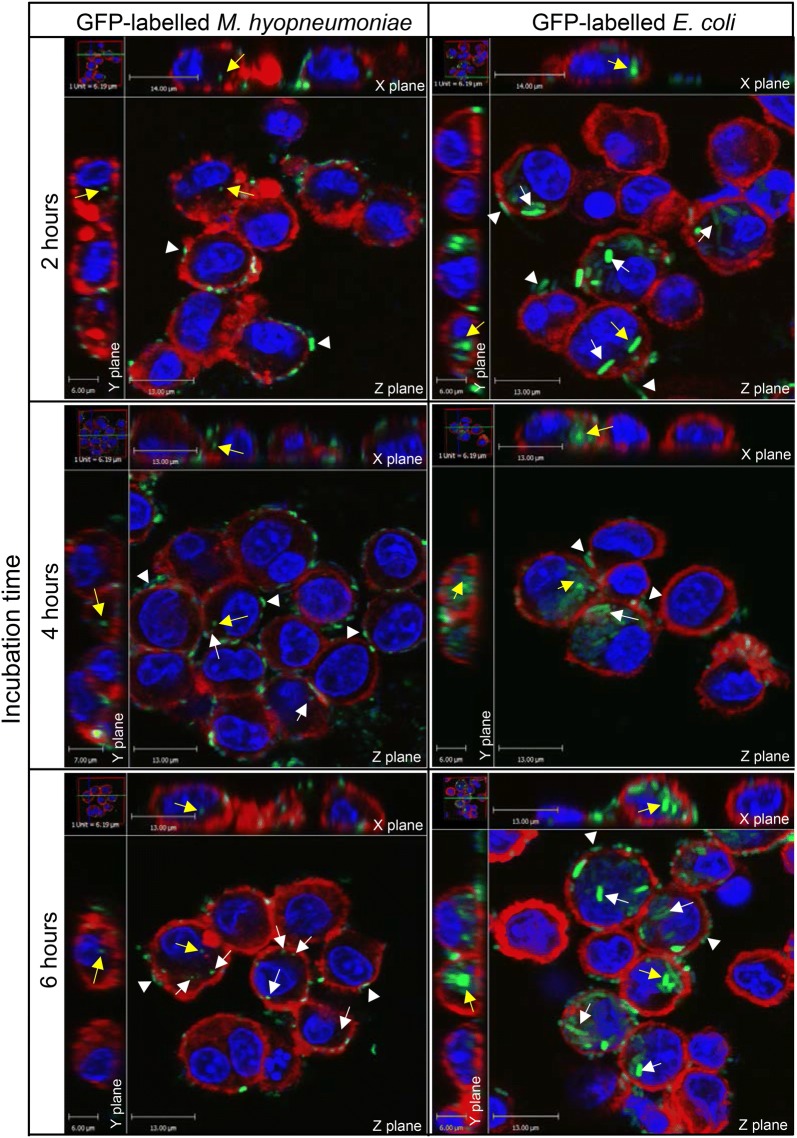
**External and internalised** ***M. hyopneumoniae*** **(pMHGFP-P97) following incubation with porcine alveolar macrophages.** Confocal micrographs of macrophages incubated with *M. hyopneumoniae* (pMHGFP-P97) or the control, *E. coli* (pKENmut2), for 2, 4 and 6 h. Fluorescent stains show: macrophage actin cytoskeleton in red, nucleic acid in blue, and GFP-labelled bacteria in green. Yellow arrows indicate internal bacteria in the XYZ planes, white arrows indicate internal bacteria, and white triangles indicate external bacteria.

*E. coli* was observed inside macrophages at each time point, demonstrating the macrophages were phagocytic (Figure 7). Macrophages exposed to *M. hyopneumoniae* (pMHGFP-P97) exhibited numerous externally-bound mycoplasma cells, many of which appeared flattened against the macrophage cell-surface (Figure 7). A few *M. hyopneumoniae* (pMHGFP-P97) were also observed inside the macrophages. An average of 2 *M. hyopneumoniae* (pMHGFP-P97) cells were found inside macrophages after 2 and 4 h of incubation (Table 2). This increased to 4 intracellular *M. hyopneumoniae* (pMHGFP-P97) after 6 h of incubation. Untransformed *M. hyopneumoniae*, visualised by nucleic acid staining, behaved similarly to GFP-labelled *M. hyopneumoniae* (data not shown).

**Table 2:** **Number of** ***M. hyopneumoniae*** **(pMHGFP-P97) inside porcine alveolar macrophages following 2, 4 and 6** **h of co-incubation**

Time (hours)	Avergae number of intracellular *M. hyopneumoniae* (pMHGFP-P97)/PAM
2	2 ± 0.4 (35 PAMs)
4	2 ± 0.6 (8 PAMs)
6	4 ± 0.5 (35 PAMs)

Standard error of the mean ± is shown, the number of porcine alveolar macrophages (PAMs) analysed is shown in parentheses.

### Opsonisation did not enhance phagocytic uptake

To assess whether opsonisation improved engulfment, *M. hyopneumoniae* (pMHGFP-P97) were exposed to three different convalescent porcine sera (sera A, B and C) or preimmune serum prior to incubation with alveolar macrophages for 2 h. The convalescent sera were sources of complement and specific antibody; preimmune serum provided complement only. Heat-inactivated foetal bovine serum was used as the negative control.

Analysis of confocal micrographs revealed that on average, the number of intracellular *M. hyopneumoniae* (pMHGFP-P97) did not differ between samples containing the different porcine sera or heat-inactivated foetal bovine serum (Table 3). It was concluded that neither complement, nor *M. hyopneumoniae*-specific serum antibody, appeared to enhance phagocytic uptake of the mycoplasma by porcine alveolar macrophages.

**Table 3:** **Effect of opsonisation on internalisation of** ***M. hyopneumoniae*** **(pMHGFP-P97) by porcine alveolar macrophages**

Serum	Average number of intracellular *M. hyopneumoniae* (pMHGFP-P97)
Preimmune	3 ± 0.5 (36 PAMs)
A	3 ± 0.8 (24 PAMs)
B	3 ± 0.5 (24 PAMs)
C	3 ± 0.3 (38 PAMs)
Heat-inactivated foetal bovine	2 ± 0.4 (35 PAMs)

Standard error of the mean ± is shown, the number of porcine alveolar macrophages (PAMs) analysed is shown in parentheses. Sera A, B and C are convalescent *M. hyopneumoniae* serum, heat inactivated bovine serum served as the control.

## Discussion

The aim of this study was to learn more about the interactions between *M. hyopneumoniae* and porcine macrophages. It was considered likely that *M. hyopneumoniae* may evade alveolar macrophage clearance mechanisms but few functional experiments investigating this have been reported. Investigations which have utilised macrophages focused primarily on cytokine production and changes in macrophage viability following exposure to *M. hyopneumoniae* or *M. hyopneumoniae*-cell fractions. For instance, Bai et al. [34] found that lipid-associated membrane proteins (LAMPs) derived from *M. hyopneumoniae* strain 232 exerted a cytopathic effect on the immortalised porcine alveolar macrophage cell line PAM 3D4/21 [34]. Although *M. hyopneumoniae* LAMPs induced apoptosis in more than 80% of PAM 3D4/21 cells, a much lesser effect was observed for whole live *M. hyopneumoniae* cells. Liu et al. [35] recently identified mhp390 (P68) as a surface membrane protein of *M. hyopneumoniae* which was able to induce caspase-3 activation, and thus apoptosis in primary porcine alveolar macrophages following 36 h of exposure.

In the present study, gentamicin survival experiments were conducted to ascertain whether viable *M. hyopneumoniae* could be recovered from inside porcine alveolar macrophages; which would suggest phagocytic uptake of this mycoplasma. The experimental results indicated very few viable *M. hyopneumoniae* were inside porcine alveolar macrophages. This could be interpreted that few *M. hyopneumoniae* had been engulfed, or that once inside macrophages *M. hyopneumoniae* were killed or few mycoplasmas remained viable. Since the macrophages appeared to engulf but not kill the control organism, *E. coli* DH5α, it was thought unlikely that *M. hyopneumoniae* had been killed by the macrophages. Therefore, it was concluded that very few *M. hyopneumoniae* had been engulfed by the macrophages. Other investigators have conducted similar experiments with different *Mycoplasma* species. Davis et al. [18] for example found that the number of inoculated *M. pulmonis* were not reduced following incubation with mouse alveolar macrophages. The investigators only observed a reduction in *M. pulmoni*s co-cultured with macrophages when hyperimmune rabbit antiserum was applied. More recent studies have indicated *M. pulmonis* resists binding to the mouse alveolar macrophages cell line, MH-S cells, which prevents phagocytic uptake and subsequent killing [19, 36]. Results from the present gentamicin survival experiments indicated *M. hyopneumoniae*, like *M. pulmonis*, evaded uptake by host alveolar macrophages; at least in the absence of opsonins. A recent publication by Raymond et al. [37] also used an in vitro gentamicin survival method to show *M. hyopneumoniae* (up to 8%) can be found intracellularly in the porcine kidney epithelial cell line, PK-15.

Since the gentamicin survival experiments relied on the viability of *M. hyopneumoniae* to inform on phagocytic uptake, an additional approach was taken that did not measure mycoplasma viability. Flow cytometric and confocal microscopy experiments were set up to provide more information about the physical interactions between *M. hyopneumoniae* and macrophages. To perform these experiments *M. hyopneumoniae* was transformed with green fluorescent protein using the extrachromosomal plasmid pMHGFP-P97, comprising *gfpmut2* controlled by the *P97* gene promoter. Two additional plasmids were investigated to express GFP in *M. hyopneumoniae*; plasmid pMHGFP2, comprising *gfpmut2* controlled by the *spiralin* gene promoter; and pMHGFP-tuf which contained *gfpmut2* controlled by the *tuf* gene promoter of *M. hyopneumoniae* strain 232. Plasmid pMHGFP-tuf failed to transform *M. hyopneumoniae* and *M. hyopneumoniae* (pMHGFP2) did not fluoresce as brightly as *M. hyopneumoniae* (pMHGFP-P97) (data not shown).

Expression of GFP in *M. hyopneumoniae* was first reported by Ishag et al. [38] who used extrachromosomal plasmid pMD18- TOgfp to express GFP. The plasmid pMD18-TOgfp possessed a *gfp* gene under control of the *P97* gene promoter sequence, and a *tetM* gene under control of an additional copy of the *P97* gene promoter. In contrast, pMHGFP-P97 produced in this study only possessed one copy of the *P97* promoter sequence to control expression of *gfpmut2*. Hence, pMHGFP-P97 may offer more stability than pMD18-TOgfp. The fluorescence intensity of *M. hyopneumoniae* (pMHGF-P97) cells was found to be dependent on time, at least 72 h of culture was required for maximal numbers of *M. hyopneumoniae* (pMHGFP-P97) cells to fluoresce. In addition, flow cytometric analysis of *M. hyopneumoniae* (pMHGFP-P97) revealed the fluorescence intensity of mycoplasma cells was heterogeneous, as indicated by the broad histogram peak. This may be related to different levels of intracellular GFP in differently aged *M. hyopneumoniae* (pMHGFP-P97) cells. Neither Ishag et al. [38] nor investigators expressing fluorescent protein in other mycoplasmas have noted a dependence of fluorescence intensity on culture time or fluorescence heterogeneity [39–41]. Despite the heterogeneous nature of *M. hyopneumoniae* (pMHGFP-P97) fluorescence, it was still suitable to use in downstream flow cytometry and confocal microscopy experiments.

Results from the flow cytometry experiments showed *M. hyopneumoniae* (pMHGFP-P97) became associated with macrophages within 1 h of co-incubation. Since the fluorescence intensities of *M. hyopneumoniae* (pMHGFP-P97)-infected macrophages treated with and without CCD were similar, it was inferred that all *M. hyopneumoniae* (pMHGFP-P97) were attached to the external surface of macrophages. An unexpected finding was that more *M. hyopneumoniae* (pMHGFP-P97) appeared bound to CCD-treated macrophages than untreated macrophages. This might be explained by an unanticipated side effect where CCD caused the macrophages to detach from the surface of the culture dish. Hence, while in suspension the CCD-treated macrophages may have encountered *M. hyopneumoniae* (pMHGFP-P97) more frequently than the adhered untreated macrophages. The confocal microscopy experiments also confirmed *M. hyopneumoniae* (pMHGFP-P97) adhered to the surface of macrophages, some mycoplasmas even appeared flattened to the macrophage cell surface. In contrast to results from the flow cytometry experiments, analysis of confocal micrographs revealed a small number of *M. hyopneumoniae* (pMHGFP-P97) were in-fact located in the cytoplasm of porcine alveolar macrophages. The slightly differing results of the confocal microscopy and flow cytometer experiments may have been due to lower sensitivity of the flow cytometer to detect internalisation of such a low number of *M. hyopneumoniae* (pMHGFP-P97). It is also noted that the gentamicin survival and flow cytometry experiments utilised macrophages originating from the same pig, whereas macrophages from another animal were used in the confocal microscopy experiments (both animals were derived from the same source at the same time). Consequently, variability in the activity of macrophages obtained from different animals should be considered. However, the phagocytic capability of both macrophage batches appeared comparable throughout this investigation, as demonstrated by engulfment of the control organism, *E. coli*, via three different methods.

It appeared that uptake of *M. hyopneumoniae* by porcine alveolar macrophages was limited and that this mycoplasma may even resist significant uptake. Powell and Clyde [42] found that in the absence of antibody *M. pneumoniae* also readily associated with the membrane of guinea pig alveolar macrophages, but were rarely found inside macrophages. The addition of hyperimmune rabbit antiserum however led to uptake of *M. pneumoniae* [42]. In the present study, the gentamicin survival, flow cytometry and initial confocal microscopy experiments had been performed in the absence of host opsonins. To address this, *M. hyopneumoniae* (pMHGFP-P97) were opsonised with porcine serum containing complement, or convalescent serum containing complement and anti-*M. hyopneumoniae* antibody. *M. hyopneumoniae* (pMHGFP-P97) were not susceptible to the bactericidal activity of serum complement, unlike the control *E. coli* (pKENmut2) which was killed by exposure to serum (data not shown). Analysis of confocal micrographs suggested serum complement did not enhance phagocytic uptake of *M. hyopneumoniae* (pMHGFP-P97), and contrary to expectation neither did the convalescent sera. It was not entirely surprising that a pathogenic organism like *M. hyopneumoniae* exhibited resistance to the bactericidal activity of complement. Although *M. hyopneumoniae* appeared resistant to complement killing, deposition of complement onto the surface of *M. hyopneumoniae* cells was not determined in this study and could not be ruled out.

*M. hyopneumoniae* convalescent serum had been purposefully excluded from some experiments to mimic the in vivo environment of the naïve host, and provide more insight into the initial interactions between *M. hyopneumoniae* and alveolar macrophages. It was surprising to find that antibody from three different convalescent sera did not promote engulfment of *M. hyopneumoniae*. Other investigators have reported swift uptake of mycoplasmas such as *M. dispar*, *M. pulmonis*, *M. ovipneumoniae*, *M. pneumoniae* and *M. bovis* by macrophages following antibody opsonisation [17, 18, 21, 22, 42]. A study by Bonnefois et al. [40] investigated uptake of fluorescently labelled *M. bovis* by monocyte-derived macrophages using confocal microscopy. The fluorescent *M. bovis* were opsonised with pooled bovine convalescent serum (ten animals), and within 30 min of co-incubation green fluorescent clumps of *M. bovis* were observed inside the macrophages. In light of these findings for *M. bovis* it was expected that convalescent serum would promote uptake of *M. hyopneumoniae* (pMHGFP-P97), but this was not the case. Although *M. hyopneumoniae* (pMHGFP-P97) were present inside macrophages, the number of intracellular mycoplasmas did not differ in the presence of naïve or convalescent serum; and intracellular *M. hyopneumoniae* (pMHGFP-P97) were located discreetly in the macrophage cytoplasm. It remains to be determined the mechanism by which the mycoplasmas entered macrophages. It was assumed that intracellular *M. hyopneumoniae* resulted from phagocytic uptake, yet a recent publication has offered a potential alternative mechanism. Raymond et al. [37] have suggested that *M. hyopneumoniae* appears to enter PK-15 cells by exploiting clathrin- and caveolae-mediated endocytosis pathways; these pathways are available in porcine alveolar macrophages [43, 44]. The intracellular *M. hyopneumoniae* were apparently able to survive fusion of endocytic vesicles with lysosomes and escape into the cell cytoplasm. So far, the authors provide only limited evidence that *M. hyopneumoniae* might invade respiratory epithelial cells in vivo [37]. However, the data presented by Raymond et al. [37] do challenge the long-held belief that *M. hyopneumoniae* is a strict extracellular pathogen.

It is not clear why convalescent sera from experimentally-infected pigs failed to promote phagocytosis of *M. hyopneumoniae* (pMHGFP-P97) in the present investigation. Two of the convalescent sera, sera B and C, were raised against heterologous strains of *M. hyopneumoniae* compared to *M. hyopneumoniae* (pMHGFP-P97) used in the present study. Hence, it is possible that strain differences prevented efficient opsonisation. However, convalescent serum A was produced following infection with *M. hyopneumoniae* strain 232, so should have contained antibody recognising surface antigens of *M. hyopneumoniae* (pMHGFP-P97). It is conceivable that the porcine host did not produce effective opsonising antibody isotypes IgG1 and IgG3 in this instance. This raises questions about the efficacy of the porcine serum response to *M. hyopneumoniae* infection. Anecdotally, production of antibody following *M. hyopneumoniae* infection can take many weeks and does not correlate well with resolution of disease. It must be noted that in this investigation the opsonising powers of mucosal antibody isotypes IgA1 and IgA2 were not examined. Experimental evidence has suggested that humoral immunity alone is not enough to overcome *M. hyopneumoniae* infection, a cell-mediated immune response is also required [8, 45, 46]. An alternative explanation may be that *M. hyopneumoniae* is preventing antibody opsonophagocytosis in some way. An antibody capture and cleavage system, designated MIB-MIP, has been identified in *M. mycoides* subsp. *capri* which may function as a virulence mechanism [47]. The authors identified potential homologues of the MIB-MIP system in other *Mycoplasma* spp. which included *M. hyopneumoniae*. The expression and functionality of a putative *M. hyopneumoniae* MIB-MIP system requires further investigation to determine whether this potential virulence mechanism aides *M. hyopneumoniae* in the evasion of humoral effector functions such as opsonophagocytosis.

In conclusion, results indicate that *M. hyopneumoniae* is not readily engulfed by primary porcine alveolar macrophages, even in the presence of convalescent porcine serum. To strengthen further this observation of an antiphagocytic property, different strains of *M. hyopneumoniae* could be evaluated following exposure to a larger panel of convalescent serum, including serum from vaccinated animals; this would also aid analysis of the opsonising power of the humoral response against *M. hyopneumoniae*. Overall, this investigation has shed more light on the interactions between *M. hyopneumoniae* and porcine alveolar macrophages, in particular it has identified a possible mechanism of immune evasion by this important pig pathogen.
